# Transverse Process Fractures in Athletes: Mechanisms, Management and Return-to-Play Considerations

**DOI:** 10.1007/s40279-026-02471-y

**Published:** 2026-06-15

**Authors:** Arnav Barve, Jake M. McDonnell, Conor McCartney, Aubrie Sowa, Danny Miller, Sean Carmody, Stacey Darwish, Joseph S. Butler

**Affiliations:** 1 School of Medicine, University College Dublin, Dublin, Ireland; 2 National Spinal Injuries Unit, Department of Trauma and Orthopaedic Surgery, Mater Misericordiae University Hospital, Dublin, Ireland; 3 Trinity Centre of Biomedical Engineering, Trinity College Dublin, Dublin, Ireland; 4 Football Association of Ireland, Irish Sports Campus, Dublin, Ireland; 5 Department of Orthopaedic Surgery and Sports Medicine, Amsterdam UMC Location University of Amsterdam, Meibergdreef 9, Amsterdam, The Netherlands; 6 Department of Orthopaedics, St. Vincent’s University Hospital, Dublin, Ireland

## Abstract

Transverse process fractures (TPFs) are typically considered minor stable spinal injuries. However, in professional athletes, these fractures can significantly impact function, training and return-to-play decisions. This narrative review examines the anatomy of transverse processes, mechanisms of injury, clinical presentation, imaging modalities, management strategies, rehabilitation principles and return-to-play considerations. A comprehensive review of the literature was conducted, incorporating case studies, sports medicine reports and trauma data to provide an evidence-based approach to TPF diagnosis and management in professional athletes. TPFs in athletes typically result from high-energy direct trauma, excessive rotational forces or muscular avulsion. While inherently stable, they may be associated with concomitant injuries, necessitating a thorough clinical and radiologic evaluation. Despite emerging imaging techniques (e.g. magnetic resonance imaging [MRI] three-dimensional [3D] T1 volumetric interpolated breath-hold examination [VIBE]), computed tomography (CT) imaging remains the gold standard for diagnosis, with MRI useful for detecting soft tissue pathology. Treatment is predominantly non-operative, focusing on multimodal pain control, early mobilization and structured rehabilitation to restore spinal function while preventing complications. Return-to-play is individualized, with most athletes resuming full activity within 3–6 weeks for isolated TPFs, though multi-level fractures or associated injuries may prolong recovery. TPFs require careful assessment and management to ensure optimal recovery and a safe return-to-play. A multidisciplinary approach involving orthopaedic surgeons, musculoskeletal radiologists, sports physicians, physiotherapists, sport scientists and coaching staff is essential to balance expedient recovery without long-term sequelae. Understanding sport-specific demands and implementing tailored rehabilitation protocols can allow athletes to make a complete recovery with minimal risk of recurrence.

## Key Points

**Table:** 

Transverse process fractures are minor injuries but can cause significant limitations in movement.
A thorough physical examination is essential to exclude associated injuries and a high index of suspicion should be maintained for any athlete with back pain.
A structured but individualised approach using the principles of PEACE&LOVE should be utilized.
Return-to-play decisions should be made on the basis of clinical findings and confirmatory imaging if required.

## Introduction

Transverse process fractures (TPFs) are injuries of the bony lateral projections of the vertebrae [1]. In the general population, isolated TPFs are considered ‘minor’ stable spine injuries [2]. They do not typically compromise spinal stability or the spinal cord because transverse processes (TPs) primarily serve as muscle and ligament attachment sites rather than weight-bearing structures. However, in the context of high-level sports, even these ostensibly minor fractures can have significant implications. Professional athletes place exceptional demands on their spines through intense contact, rotation and extension; so a TPF can cause considerable pain and functional limitation despite being a stable injury [3, 4]. Moreover, while the prognosis is generally favourable, the injury must be managed carefully to minimize time away from play and avoid complications. This narrative review details the anatomy of TPs, mechanisms of injury in sports, associated injuries, clinical assessment, imaging, management principles, rehabilitation, risks and return-to-play (RTP) considerations in professional athletes with TPFs. A thorough review of the literature surrounding this topic was conducted using PubMed, Embase and Google Scholar. Studies related to the anatomical features, mechanisms of injury, diagnostic protocols and management principles of TPFs were included and carefully examined. The findings from all relevant studies were compiled and this thorough review was drafted, with specific subheadings for each aspect of this injury.

## Anatomy of Transverse Processes and Athlete-Specific Considerations

Each vertebra has two TPs extending laterally from the junction of the pedicle and lamina [1]. Their primary role is to serve as attachment points for paraspinal muscles and ligaments. For example, in the lumbar spine the TPs are relatively large and robust, providing attachments for major trunk muscles such as the quadratus lumborum and psoas major [5]. In the thoracic spine, TPs articulate with the ribs at the costotransverse joints, anchoring rib motion during breathing. Cervical TPs are unique in containing the transverse foramina, through which the vertebral arteries pass. These regional anatomic differences mean that the consequences of a TPF can vary by location. A lumbar TPF often involves attached muscle avulsion or hematoma, whereas a thoracic TPF may coincide with rib injury, and a cervical TPF raises concern for vertebral artery injury [6–9]. Importantly, because TPs are lateral to the spinal canal, isolated TPFs rarely cause neurological deficits or spinal instability [10]. This inherent stability underlies why these injuries, when truly isolated, are typically managed non-operatively and have a good prognosis.

## Prognosis in Professional Athletes

In elite athletes, the overall prognosis for isolated TPFs is generally excellent, with most returning fully to sport after a period of offload and progressive rehabilitation [11–14]. TPFs tend to heal without long-term sequelae aside from potential localized soreness or muscle stiffness. A seminal study by Tewes et al. of 29 National Football League (NFL; American Football) players with lumbar TPFs found an average RTP time of only ~ 3.5 weeks with minimal long-term sequelae [12]. Similarly, a case report by Brynin et al., of a high-school American Football player who sustained lumbar TPFs (L2 and L3) from a helmet impact, demonstrated full recovery and RTP at 4 weeks post-injury [13].

The prognosis can be worse if TPFs are accompanied by other injuries or if multiple adjacent TPs are fractured. Athletes with multi-level TPFs tend to report higher initial pain and disability. For example, a case report by Bali et al., regarding a professional cricket player who sustained stress fractures of the left L1–L5 TPs, initially reported extreme pain which interfered with his activities of daily living and required a prolonged recovery period of 9–10 months [3]. Overall, because TPFs do not destabilize the spine, prognosis is mainly determined by soft tissue healing and pain control. The major challenges are managing pain and ensuring a safe return without re-injury.

## Sport- and Position-Specific Considerations

The functional impact of a TPF can vary with the sport and the athlete’s position/role. In sports that demand heavy contact (e.g. American Football, rugby), players often wear protective gear (flak jackets, padded vests) after a lumbar TPF to shield the area upon RTP [12, 13]. Tewes et al. noted that most players returning from TPF wore such padding, and only one suffered a recurrent fracture [12]. This suggests that while padding may help, it cannot guarantee prevention of re-injury in collision sports and may negatively affect athlete performance owing to its cumbersome nature. Position-wise, athletes who frequently rotate or extend their trunk (quarterbacks throwing, golfers swinging, fast bowlers, gymnasts, etc.) might experience more functional limitation from a TPF owing to pain with those motions. For instance, in the case of Bali et al., fast bowling with a mixed-type bowling action exposed the athlete to repetitive rotational stresses which caused multiple lumbar TPFs [3]. Each sport may also entail different rates of healing and risks upon return. In low intensity contact sports, such as soccer (football) or field hockey, which involve less contact rates as compared with NFL/rugby with higher collision rates, a player may tolerate a healing TPF relatively well once acute pain subsides. Furthermore, a soccer player needs full trunk flexibility for kicking and hence may require near-complete healing before high-level play, whereas a taped-up hockey player might participate earlier if they can skate with minimal discomfort. Thus, clinicians should tailor prognostic expectations and RTP decisions to the specific athletic activity, ensuring that sport-specific motions (twisting, tackling, throwing, etc.) can be performed safely and effectively.

## Mechanisms of Injury in Sports

### Causes of Acute and Chronic Trauma

In professional sports, TPFs can result from both acute high-energy mechanisms and repetitive stress. Acutely, a direct impact to the flank or back is a common cause. Contact sports athletes may incur TPFs after experiencing direct impact to the body, either from the opponent or by falling on the ground. Tewes et al. and Brynin et al. both reported direct impact to the back as a major cause of TPFs [12, 13]. Falls with impact are another significant mechanism in sports such as soccer and horse riding [14–16]. Thus, a high index of suspicion for TPFs should be maintained in soccer players who have suffered an impact-related injury during gameplay. Studies have shown that various upper trunk, lower trunk and pelvic muscles are utilized during soccer-related movements, and their sudden contraction has the potential to cause TPFs [17, 18]. The mechanism of this injury is similar to the mechanism of a clay-shoveler’s fracture or a lateral malleolar fracture, where the bony segment is avulsed due to excessive muscular force [19, 20].

Repetitive stress can also produce TPFs, causing minor trauma to the bony segment, ultimately resulting in fragmentation. The literature also noted that repetitive microtrauma to the TP may increase the likelihood of developing stress fractures. A case report regarding an elite rower by McGuire et al. showed that in the absence of any direct trauma to the back, a single level TPF developed [11]. The cause of the fracture was postulated to be an asymmetric increase in the muscular load exerted on a weakened L3 TP. Similarly, in the report by Bali et al., multiple lumbar TPFs were seen without a single acute event [3]. The chronic repetitive extension and rotation during bowling likely led to microfractures accumulating. Similarly, baseball pitchers or tennis players could theoretically develop a TP stress fracture from repetitive trunk rotation, though documented cases are scarce.

In summary, TPFs can be the result of direct trauma, repetitive use or violent muscle action, due to either a strong concentric muscle contraction or significant muscle lengthening.

### Examples by Sport

**Fig. 1:**
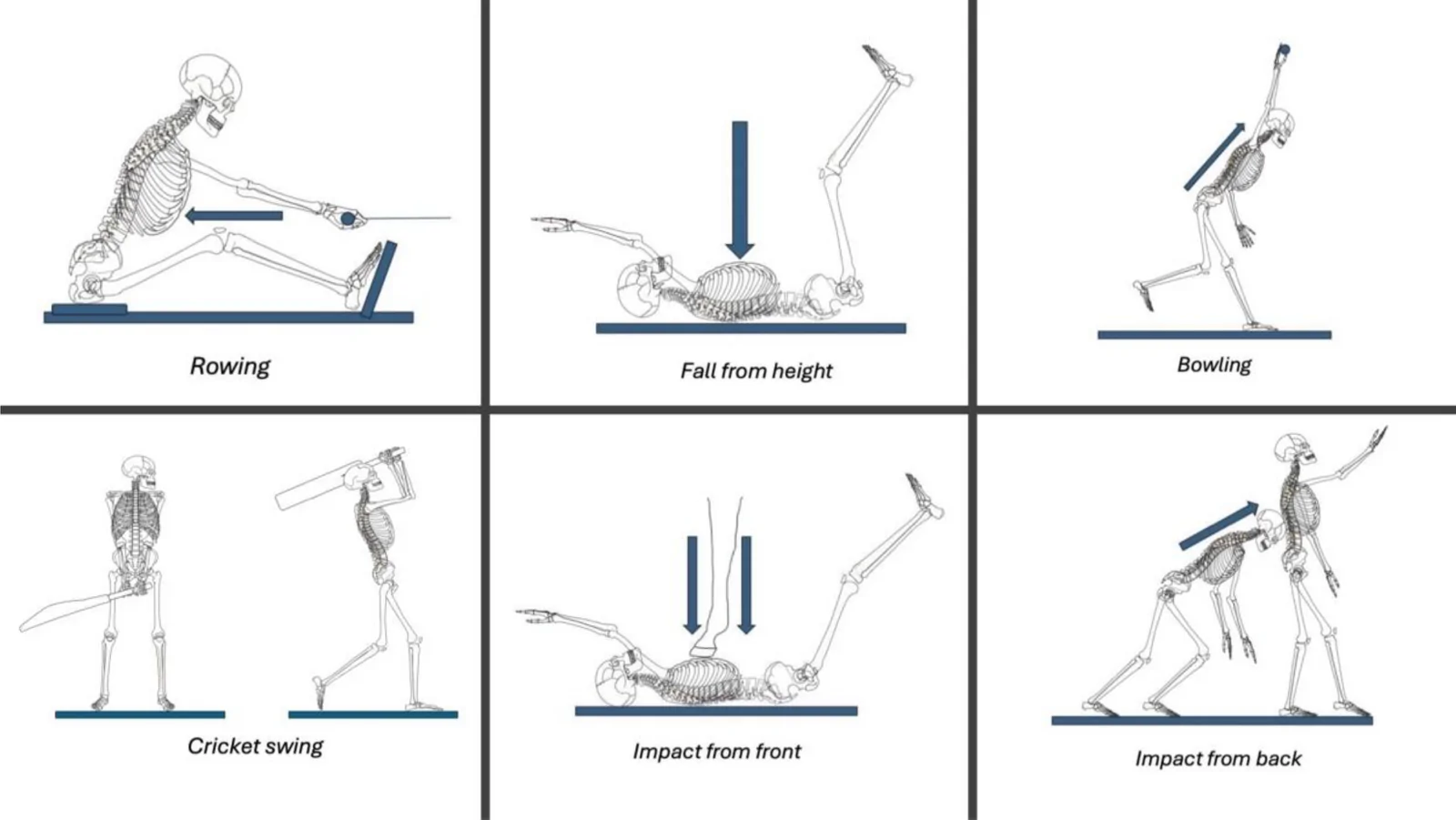
Different sports-related mechanisms for transvere process fractures [21]

To illustrate, American Football tends to produce TPFs via direct collisions [12]. By this mechanism, rugby players, Australian Rules football, Gaelic Football players and those who play hurling can also be considered a high-risk group for developing TPFs from direct trauma. Soccer players can incur TPFs in aerial challenges or falls; the Premier League player in Gray et al. fell from a height while performing an overhead kick [14]. Combat sports (mixed martial arts [MMA], judo) may also give rise to TPFs when fighters are thrown onto their side or take a direct blow to the mid-back. Equestrian and motor sports frequently involve high-G lateral impacts that can lead to fractured TPs as part of complex injury patterns [15]. Gymnasts or pole vaulters who land awkwardly might also suffer isolated TPFs if the flank hits the apparatus or ground forcefully. Even non-contact athletes can sustain TPFs through unique mechanisms: a golfer or batter could theoretically avulse a TP during a forceful swing if combined with a sudden jarring stop. Table 1 presents selected reported cases/series of TPFs in athletes with their mechanisms and outcomes. These examples reinforce the idea that the three main mechanisms involved in the development of TPFs are direct contact, excessive spinal motion and repetitive spinal movements. Awareness of the likely mechanism can also guide clinicians in their evaluation. The occurrence of these fractures through different sports-related mechanisms has been outlined in Fig. 1.

**Table 1:** Reported TPFs in athletes—mechanisms and outcomes

Reference Number	Article	Year of Publication	Study type	Geography	Cohort size (*n*)	Sport involved	Mechanism of injury	Number of levels injured	Time to return-to-play
[64]	Kiel et al	2025	Book chapter	USA	–	–	Repetitive stress	–	–
[11]	McGuire et al	2020	Case report	USA	1	Rowing	Stress fracture of L3, caused by an asymmetric increase in the muscular load on her L3 TP	1 (L3)	13 weeks
[14]	Gray et al	2015	Case report	UK	1	Soccer	Fall from height, landing on left side and lower back	2 (L2, L3)	68 days (~ 10 weeks)
[15]	Srinivasan et al	2014	Retrospective review	USA	80 (8 patients had spinal fractures, of which 63% were TPFs)	Equestrian	Kicked = 39 (49%), fall = 22 (28%), stepped on = 4 (5%), horse fell on patient = 13 (16%), not involving horse = 1 (1%), other = 1 (1%)	–	–
[7]	Gertzbein et al	2012	Retrospective review	USA	119 (of which 14 skiers and 10 snowboarders had isolated TPF)	Skiing and Snowboarding	Authors state that TPFs occur when the spine strikes the ground, which causes an intense psoas muscle spasm and the subsequent avulsion of the bony process	1	–
[3]	Bali et al	2011	Case report	India	1	Cricket	A mixed-type bowling action, which involves repeated hyperextension and lateral flexion of the spine	5 (L1–L5)	9–10 months
[13]	Brynin et al	2001	Case report	USA	1	American Football	Direct impact (spearing) to the right side of the lower back	2 (L2, L3)	4 weeks
[12]	Tewes et al	1995	Retrospective review	USA	29	American Football	Direct impact = 27 (93%), torsion = 2 (7%)	1 level = 12 (41%), 2 levels = 7 (24%), 3 levels = 8 (28%)	Mean = 3.5 weeks (1 level = 16 days, 2 levels = 19 days, 3 levels = 36 days

*L* lumbar, *TP* transverse process, *TPF(s)* transverse process fractures(s)

**Fig. 2:**
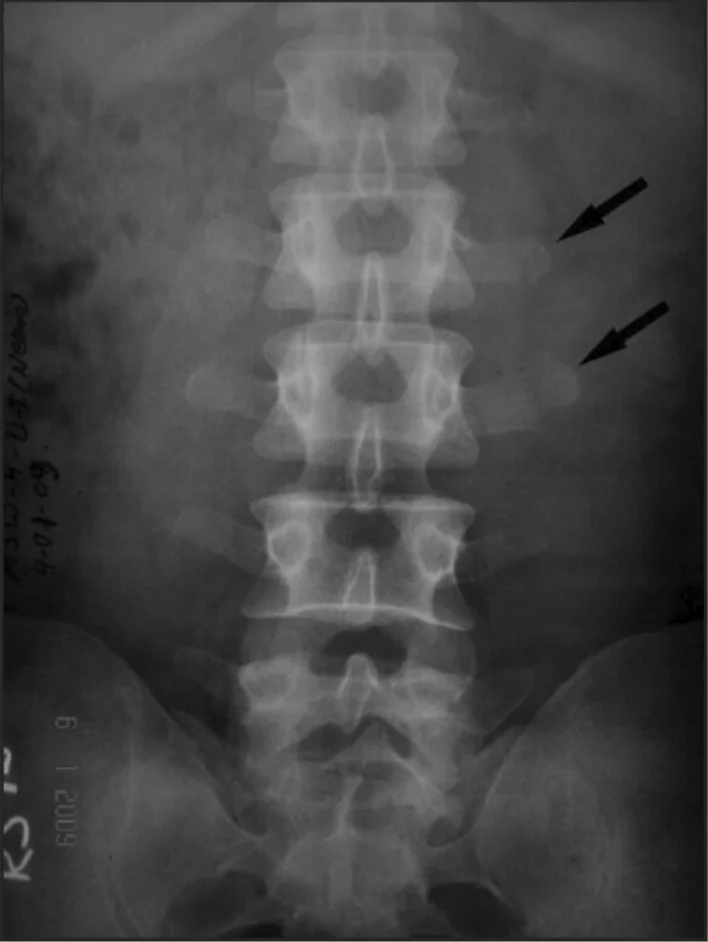
Plain film radiograph of the lumbar spine showing fractures of the left L2 and L3 TPs [22]; reproduced from Agrawal et al. [22], with permission

**Fig. 3:**
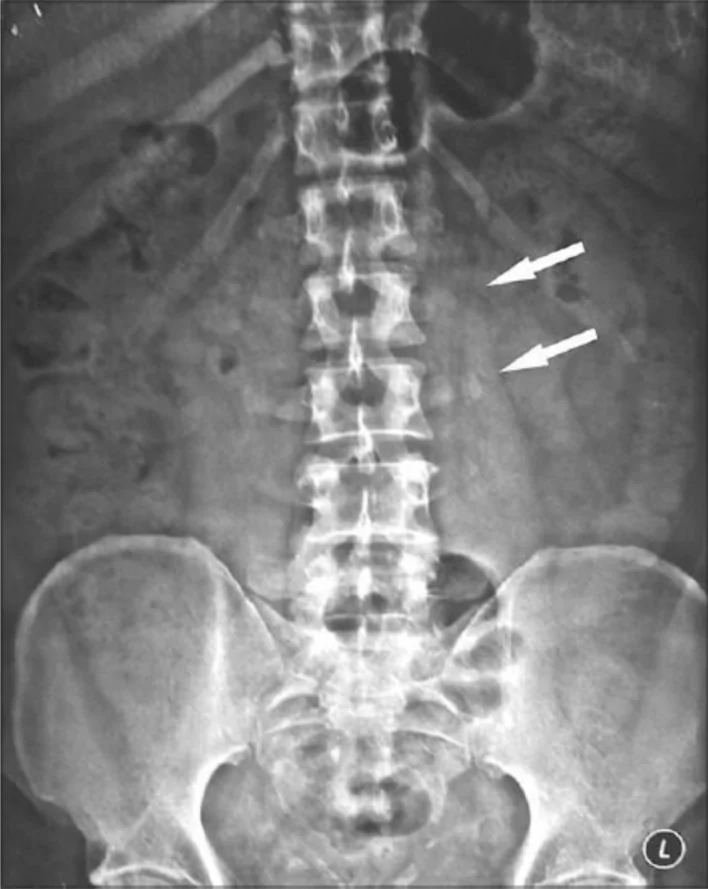
Plain film radiograph of the abdomen and pelvis showing the artefacts masking the fracture line [23]; reproduced from Hegde et al. [23], with permission

**Fig. 4:**
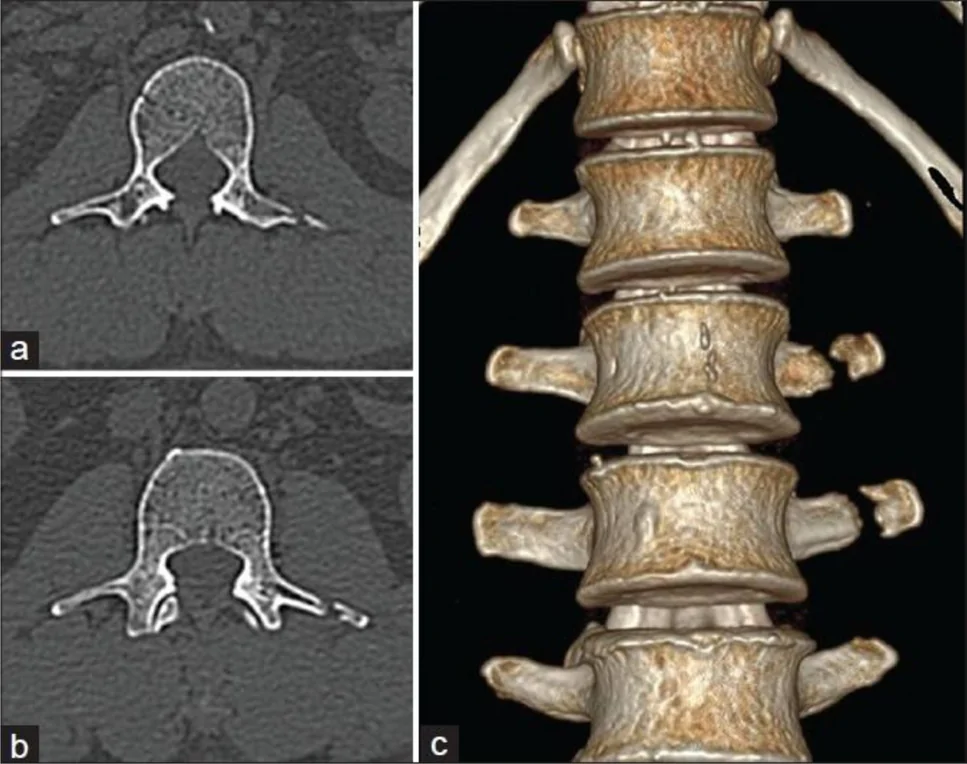
(**a**) and (**b**) Helical CT scans with (**c**) 3D reconstruction showing the fracture segments [23]; reproduced from Hegde et al. [23], with permission

Numerous studies have shown the occurrence of transverse process fractures through the different mechanisms outlined in the illustration above. They underline the common predictable injury mechanisms which can lead to TPFs. Thus, clinicians should maintain a high index of suspicion for TPFs when a patient outlines a similar injury mechanism. In the study by Agrawal et al., a 40-year-old man suffered left L2–3 TPFs after falling from a height of 10 feet, which could be visualised on plain film radiographs of the abdomen (Fig. 2) [22]. However, another study by Hegde et al. underlined the importance of computed tomography (CT) imaging in the diagnosis of TPFs [23]. The study outlined the case of a 40-year-old patient who sustained a high-energy injury in a road traffic accident (RTA). The authors noted that the TPF suffered by this patient was initially not evident on the plain radiograph of the abdomen and pelvis since it was masked by abdominal artefacts (Fig. 3). However, a helical CT scan with 3D reconstruction clearly showed the fracture fragments (Fig. 4).

## Associated Injuries and Implications

Although TPFs by themselves are stable, their presence often signifies that substantial force was transmitted to the body. In trauma literature, lumbar TPFs are considered a ‘sentinel marker’ of high-energy trauma, often accompanied by injuries to the abdomen, heart, liver, spleen, kidneys, ribs and pelvis [8, 24, 25]. In the sports setting, the energy of injury is generally lower than a road traffic collision, and accordingly severe associated injuries are less common. Nevertheless, they do occur, especially in collision sports. In the case of Dutson et al., a soccer player suffered a traumatic transverse colon rupture along with a TPF, requiring surgical repair [26]. This rare but serious associated injury resulted from a knee-to-flank impact that fractured the TP. Instead of lacerations, the impact can often cause contusions, as reported by Tewes et al. [12].

### Common Concurrent Injuries

The common concurrent injuries associated with TPFs can be divided into focal or remote. Locally, disruption of the transverse foramen in cervical TPFs has been shown to cause concomitant vertebral artery injury (VAI), such as dissection or occlusion [9]. Thus, athletes who sustain cervical TPFs—common in wrestling or hockey—should be assessed for vertebral artery insufficiency, which may present as dizziness or nystagmus. Injuries to the vertebral column should also be considered when a TPF is present, as their occurrence with TPFs has been noted in the literature [27]. Hence, any athlete presenting with midline spinal tenderness or neurological symptoms should undergo further evaluation to assess for associated vertebral injuries. This can be done through novel magnetic resonance imaging (MRI) sequences (e.g. volumetric interpolated breath-hold examination [VIBE]) to reduce the exposure to ionising radiation (e.g. CT, X-ray). Muscle and soft tissue injuries are also frequently associated with TPFs due to the strong muscular attachments at these sites [28]. These can hinder radiological diagnosis, irritate adjacent structures and ultimately prolong recovery and require careful rehabilitation [29, 30]. Moreover, since TPFs are usually not associated with neurological symptoms, any persistent neurological deficits should prompt thorough evaluation for additional pathologies.

TPFs can also present with injuries to the thorax and abdomen. Rib, splenic and hepatic injuries are particularly common with thoracic or upper lumbar TPFs, as the same impact that fractures a TP may also injure adjacent structures [8]. This is frequently observed in athletes who sustain flank trauma. Renal injuries are another significant concern owing to the anatomical proximity of the kidneys to the T12–L3 TPs [12]. These injuries, which often present as microscopic haematuria following a flank impact, can be associated with TPFs [24, 31]. While they are rarer in sports, their impact is far-reaching and should thus be assessed for with a thorough abdominal examination, focused assessment with sonography in trauma (FAST) ultrasound or CT imaging.

### Implications for Prognosis and Recovery

When associated injuries are present, they often dictate the initial management and lengthen the athlete’s time to RTP. An isolated TPF might only need analgesia and a period of offload. However, multiple TPFs or associated visceral injuries may need restricted activity until all injuries heal. In the case of Dutson et al., the occurrence of a transverse colon rupture significantly increased the recovery period to beyond 12 weeks [26]. Even less severe concomitant injuries like rib fractures will amplify pain and could necessitate additional analgesia and a longer offload period. Therefore, the first step in managing an athlete with a TPF is to ‘clear’ the possibility of associated injuries by performing imaging of the abdomen/pelvis if warranted, evaluating the entire spine and monitoring for any signs of internal injury over the first 24–48 h. If none are present, the prognosis is typically favourable. If they are present, appropriate specialists should be involved to determine the next steps.

## Clinical Presentation and Assessment

### Signs and Symptoms

Athletes sustaining a TFP commonly present with acute, localized pain on one side of the spine, typically described as sharp or stabbing in nature. The severity often forces the athlete to stop play immediately [13, 14]. Muscle spasms in the paraspinal muscles are frequently observed, as the body attempts to splint the injured area. These spasms may restrict trunk movement and, over time, could even result in a compensatory scoliotic posture [32]. Anecdotal reports from experienced physiotherapists suggest that early compensatory movements—particularly lateral flexion toward the painful side—may predispose athletes to contralateral avulsion injuries. Athletes often have trouble bending away from the injured side or rotating the trunk. Pain may intensify with deep breathing, coughing or sneezing, and may radiate towards the flank or abdomen, mimicking thoracic or abdominal pathology. However, unlike visceral pain, TPF pain is well-localized, worsens with movement and generally improves with rest.

On physical examination, the hallmark finding is point tenderness over the affected transverse processes, along with pain-limited spinal movements, including flexion, extension and lateral flexion [11, 13]. This is best assessed with the patient prone or standing, with palpation approximately 5–8 cm lateral to the midline on the symptomatic side, over the TP region. In the lumbar spine, this area lies between the paraspinal muscles and the lateral abdominal wall. While surface deformities and ecchymosis are uncommon, they may be seen in more extensive injuries. Paraspinal muscle spasm is often palpable as firmness or protective guarding [11]. Neurological examination is typically normal in isolated TPFs, as the spinal cord and nerve roots are not involved. However, any neurological deficit should prompt investigation for more complex injuries.

### Assessment and Differential Diagnosis

Clinical assessment of suspected TPFs requires a high index of suspicion and a thorough differential diagnosis. Muscle contusions, commonly coexisting with TPFs, may mimic symptoms and should hence be assessed for using advanced imaging techniques which enable visualisation of cortical disruption or soft tissue oedema [30]. Pain radiating to the flank, worsened by deep breathing, may suggest rib fractures, pulmonary contusions, or even pneumothorax [33]. These possibilities should be ruled out with chest palpation, auscultation and potentially a chest X-ray.

Vertebral body fractures typically present with midline rather than lateral pain and are usually associated with axial loading mechanisms, such as falls from height or direct vertical impact [34]. These may present with kyphotic deformities or palpable step-offs. Radicular symptoms or mild neurological deficits suggest potential involvement of the facet joints or dislocations, necessitating urgent imaging [35].

Importantly, TPFs may also coincide with visceral injuries. Symptoms such as diffuse abdominal pain, peritoneal signs (guarding, rigidity), or hypotension should prompt a detailed abdominal examination [36]. Therefore, a comprehensive clinical evaluation should include palpation of the entire spine, costovertebral angle tenderness and abdominal assessment. While no single test confirms a TPF, useful clinical clues include lateralized pain with movement, focal tenderness over the TP area and pain with trunk rotation or side bending. In high-energy trauma scenarios, evaluation should follow advanced trauma life support (ATLS) principles to first ensure haemodynamic stability before focusing on spinal or abdominal findings.

### On-Field and Pitch-Side Assessment

TPFs often manifest as localized flank pain without neurological signs, allowing some athletes to walk off the field with assistance. However, an antalgic gait may be present. On-field assessment should prioritize excluding spinal cord or visceral injury using structured trauma protocols such as danger, response, catastrophic haemorrhage, airway, C-spine, breathing, circulation, disability, expose and examine (DRCA(c)BCDE). If neurological signs are absent and the athlete is stable, further evaluation can proceed in detail.

A structured and objective pitch-side assessment is vital to avoid misdiagnosis. Brynin et al. highlighted a case where the TPF was initially missed, leading to potentially harmful treatment [13]. Thus, clinicians should maintain high suspicion in athletes with lateral back pain following impact or extreme motion, particularly when accompanied by point tenderness and restricted range of motion. Importantly, neurological deficits should not be present in isolated TPFs. Prompt identification enables timely imaging and prevents premature RTP or inappropriate interventions.

### Summary

Clinicians should suspect a TPF when an athlete presents with localized, movement-exacerbated spinal pain post-trauma, point tenderness over the transverse process, limited but intact range of motion and no neurological signs. A high index of suspicion, particularly with high-energy mechanisms, facilitates early diagnosis and safe management.

## Imaging and the Role of Serial Imaging

### Indications for Imaging

Imaging should promptly be obtained in any athlete with suspected TPFs or undifferentiated, significant back pain following trauma. Owing to the potential for concurrent injuries and the subtlety of TPF presentations, clinicians should maintain a low threshold for imaging. Although plain radiographs are commonly used as a first-line investigations, their sensitivity is limited. An anteroposterior (AP) lumbar spine X-ray may occasionally reveal a vertically or obliquely oriented TPF, typically seen as an avulsed tip at the base of the transverse process [13, 29]. However, many TPFs are missed on plain films, with some reports suggesting that up to 61% of lumbar TPFs visible on CT were not detected on initial radiographs [37]. Factors such as overlying anatomical structures and minimal displacement contribute to this. Subtle radiographic signs, including loss of the psoas shadow or a small avulsion fragment, may be the only clue [29]. If clinical suspicion remains high despite normal or inconclusive radiographs, advanced imaging should follow without delay.

### Computed Tomography

CT remains the gold standard for diagnosing TPFs. Thin-slice CT offers excellent resolution, allowing for detection of even minor fractures. In trauma settings, CT scans of the abdomen and pelvis frequently identify lumbar TPFs incidentally during evaluations for visceral injury [37]. For athletes, a focused spinal CT provides detailed information on the number, exact location and displacement of fractured transverse processes, as well as extension into adjacent structures like the pedicle or facet. In a study by Gray et al., an MRI initially revealed bone oedema at L2–L3, and subsequent CT on day two confirmed the fracture [14]. Given its rapid acquisition and high sensitivity, CT is often preferred when a significant spinal injury is suspected. In polytrauma cases, whole-body trauma CT protocols can also detect TPFs. However, radiation exposure remains a key limitation in sports medicine, especially if serial imaging is anticipated for monitoring recovery and RTP decisions.

### Magnetic Resonance Imaging

**Fig. 5:**
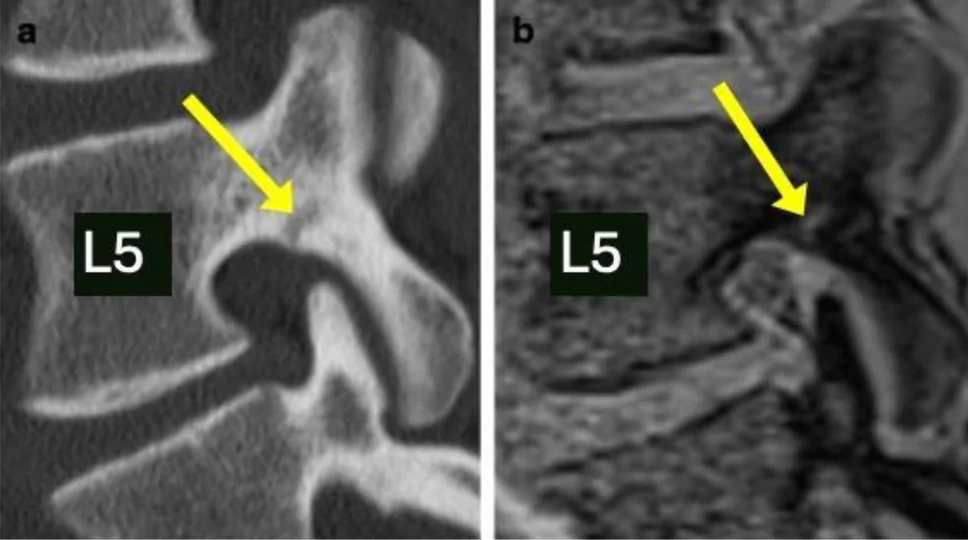
(**a**) Sagittal CT of L5 and (**b**) sagittal 3-T MRI T-1 VIBE (volumetric interpolated breath-hold examination) sequence both demonstrating pars fracture at L5 (fracture indicated by yellow arrows) [39]; reproduced from Ang et al. [39], with permission

**Fig. 6:**
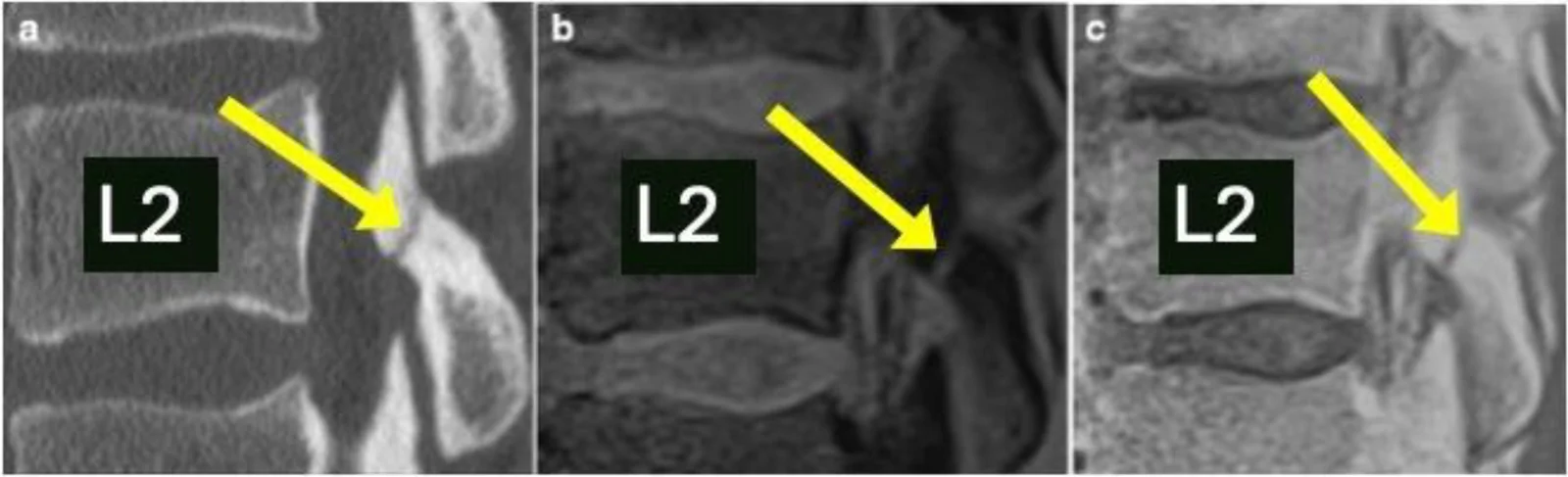
(**a**) Sagittal CT of L2 and (**b**) sagittal 3-T MRI T-1 VIBE (volumetric interpolated breath-hold examination) sequence both demonstrating complete pars fracture at L2. (**c**) Inverted sagittal 3-T MRI T-1 VIBE (volumetric interpolated breath-hold examination) sequence to simulate a CT style appearance (fracture indicated by yellow arrows) [39]; reproduced from Ang et al. [39], with permission

**Fig. 7:**
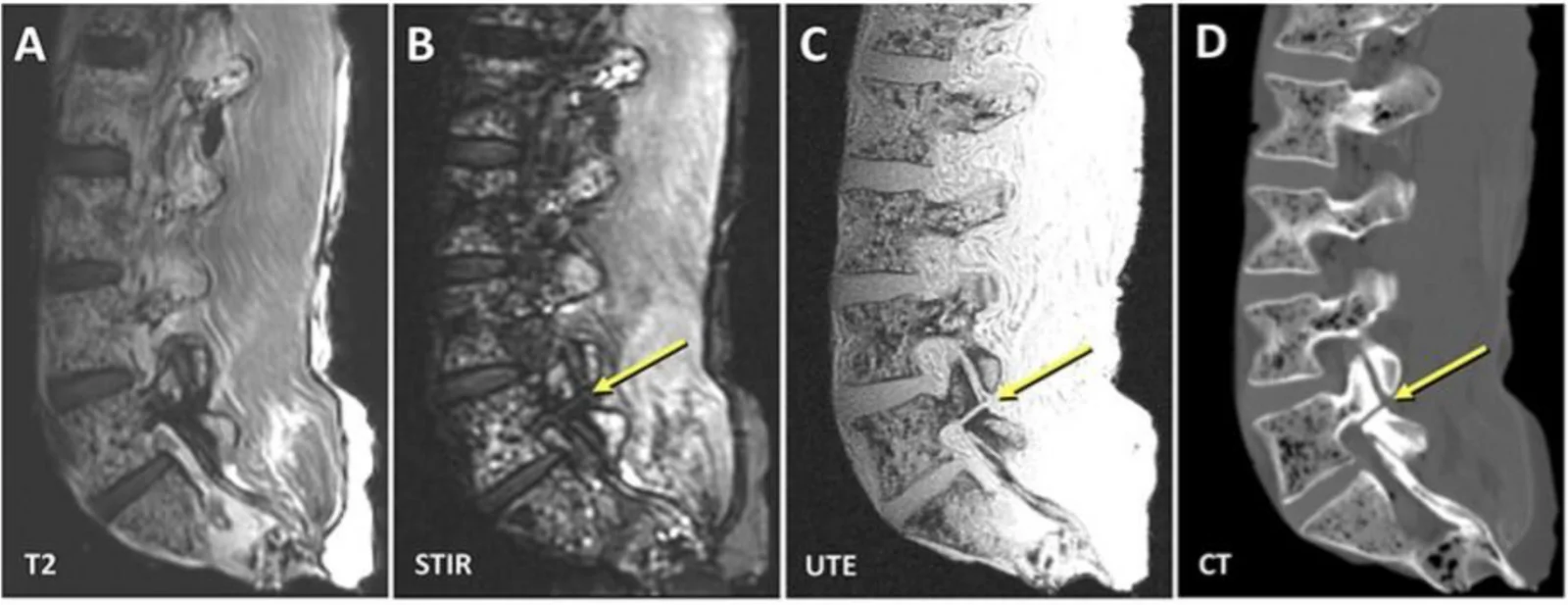
A 42-year-old female with pars fracture not detected on (**A**) conventional T2 imaging. The short tau inversion recovery (STIR) sequence (**B**) shows pars defect to some extent. Both the ultrashort time-to-echo (UTE) (**C**) and the CT (**D**) images clearly depict fracture within ~ 1 mm width (fracture indicated by yellow arrows) [42]; reproduced from Finkenstaedt et al. [42], with permission

While MRI is less sensitive than CT for detecting fine cortical disruptions, it excels in visualising bone marrow oedema, soft tissue injury and neural involvement [38]. MRI is particularly useful for identifying stress reactions, distinguishing acute from chronic fractures and assessing associated disc, ligamentous or nerve injuries. For example, Brynin et al. described X-rays suggestive of TPFs that were later confirmed via CT; an earlier MRI could have detected oedema, facilitating earlier management [13]. In the case of Gray et al., an MRI was prioritized, presumably to evaluate nerve integrity [14]. Emerging MRI techniques have shown promise in detecting subtle and diagnostically challenging vertebral fractures, particularly pars fractures. Techniques such as 3-T MRI with thin-slice 3D T1 VIBE sequences have shown 100% accuracy in detecting complete pars fractures initially diagnosed by CT (Figs. 5 and 6) [39]. Furthermore, innovations such as short tau inversion recovery (STIR) and ultrashort time-to-echo (UTE) MRI sequences enhance diagnostic accuracy: STIR helps detect osteoporotic fractures missed by CT, while UTE enables direct visualization of cortical bone, offering insights into bone quality (Fig. 7) [40, 41].  Hence, applying these findings and using these modalities for the detection of TPFs  has the potential to reduce radiation exposure and enhance diagnostic yield, offering a comprehensive, safer imaging approach.

**Fig. 8:**
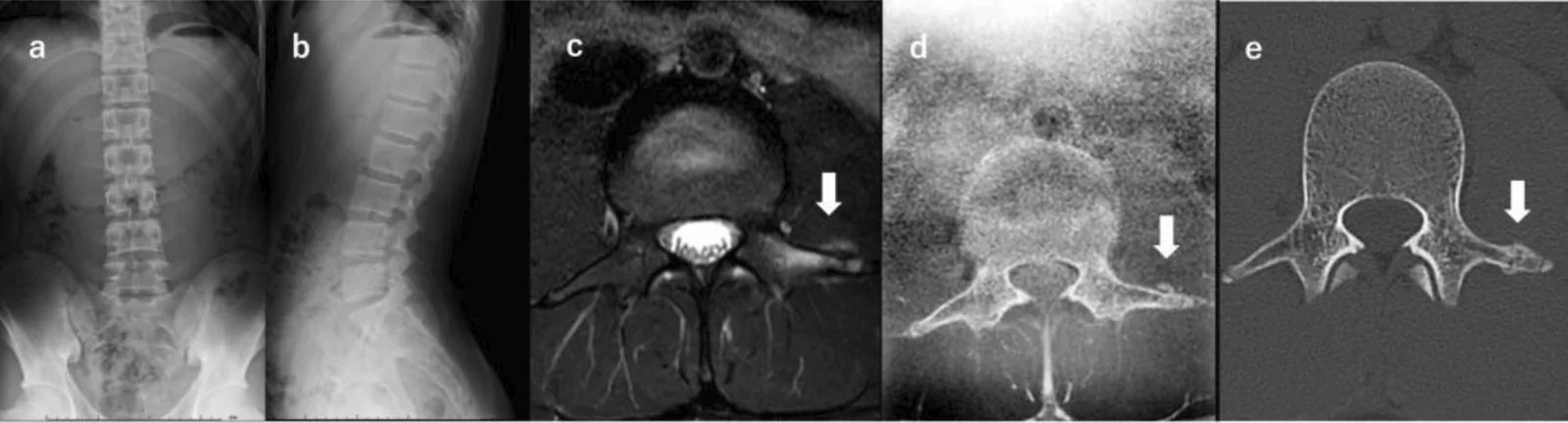
Sequential imaging findings of an L3 transverse process fracture in a 16-year-old male high school American Football player [46]; reproduced from Minami et al. [46], with permission

**Fig. 9:**
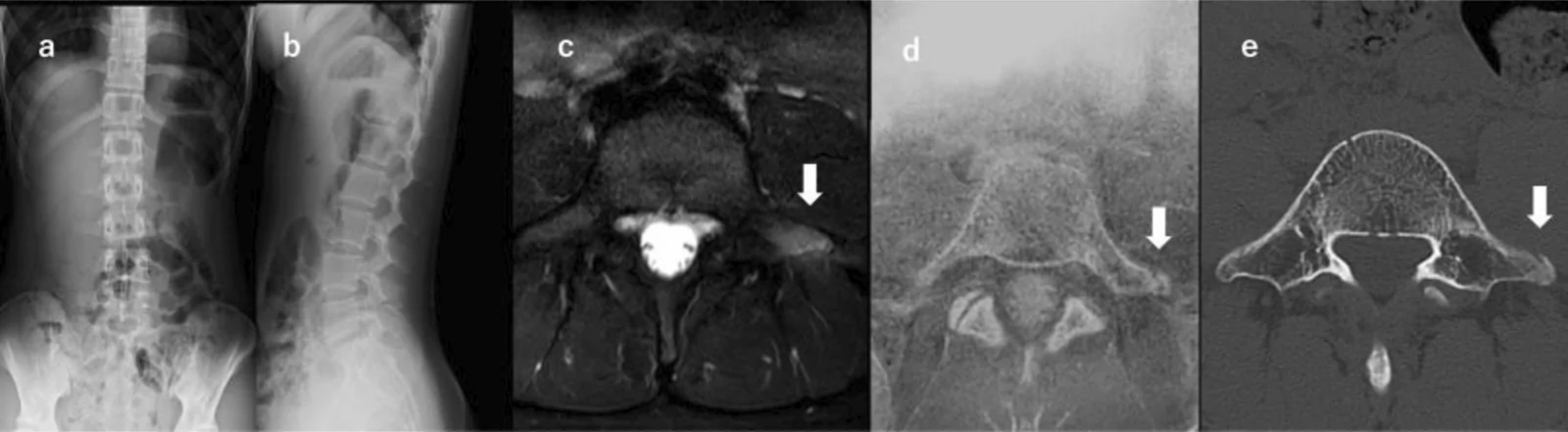
Sequential imaging findings of an L5 transverse process fracture in a 15-year-old female junior high school ballet dancer [46]; reproduced from Minami et al. [46], with permission

A central question in athlete management is whether follow-up imaging is necessary before RTP. While no consensus exists, clinical resolution—absence of pain and restored function—is often sufficient for clearance, as TPFs are stable and typically managed non-operatively [11]. Persistent pain, multi-level TPFs, cervical spine involvement, associated vertebral fractures requiring surgery or assessment of fracture union may warrant repeat imaging to confirm healing or exclude new issues [3, 43–45]. Inconclusive initial scans may also necessitate repeat imaging [13]. Some teams employ single-photon emission computed tomography (SPECT) to differentiate active from healed fractures [47]. Increasingly, elite teams use confirmatory CT or MRI before return to contact sport [48]. A study by Minami et al. outlined this imaging protocol for the treatment of two adolescent athletes with stress fractures of the lumbar TPs [46]. Both athletes had plain radiographs which showed no abnormalities, axial STIR MRIs showing high signal intensity at the fracture site, MRI bone imaging revealing the fracture line, and an axial CT scan at four weeks confirming bone union. The images for these athletes are outlined in Figs. 8 and 9. This sequential imaging protocol highlights the utility of different imaging modalities in the diagnostic and recovery period of TPFs.

## Principles of Management

The management of TPFs in athletes is predominantly non-operative, focusing on symptom control and functional rehabilitation, with surgical intervention rarely required. Core principles include protecting the athlete from further harm (particularly in the presence of associated injuries), relieving pain to facilitate movement and respiration and supporting the natural healing of bone and surrounding soft tissue.

### Initial Management and Pain Control

During the acute phase, rest and pain control are the primary interventions. Athletes with a suspected or confirmed TPF should be temporarily removed from training and match-play until appropriate investigations are completed. Unlike unstable spinal injuries, routine bracing is not typically necessary for isolated TPFs [49]. Early mobilization is encouraged, as prolonged immobilization may lead to deconditioning without conferring additional benefit in stable fractures.

Pain management should follow a structured, stepwise approach. First-line treatment includes oral analgesics such as non-steroidal anti-inflammatory drugs (NSAIDs; e.g. ibuprofen) and paracetamol [14]. Topical treatments, including NSAID gels or lidocaine patches, may offer additional localized relief [50, 51]. However, NSAIDs should be used cautiously in line with the keeping-SCORE treatment recommendations, given their potential to delay tissue healing and cause adverse effects [52, 53]. When NSAIDs are contraindicated or inadequate, paracetamol in combination with mild opioids such as codeine can be used. Tramadol, however, is prohibited for use in athletes under World Anti-Doping Agency (WADA) regulations and should be avoided if possible [54].

Muscle spasm, a common contributor to TPF pain, may be treated with benzodiazepines like diazepam, though these require caution owing to sedative effects and risk of dependency [55]. In cases of intense, localized pain—especially when rib fractures coexist—regional anaesthetic techniques, such as paravertebral or erector spinae plane blocks, may be considered. These target the dorsal rami near the transverse process to alleviate pain and improve breathing [33, 57].

Adopting the protect, elevate, avoid anti-inflammatory modalities, compress, educate (PEACE) and load, optimism, vascularisation, exercise (LOVE) principles has proven beneficial in managing soft-tissue injuries associated with TPFs, integrating both acute and rehabilitation strategies [58].

### Role of Bracing

Rigid bracing, including thoracolumbar sacral orthoses (TLSO), is generally unnecessary for thoracolumbar TPFs. Research indicates bracing does not significantly lower reinjury rates and may lead to complications such as skin irritation or discomfort [12, 49]. A soft binder or corset may be used for short-term comfort but remains controversial in the literature [59]. For cervical TPFs, a soft collar may be prescribed for 2–4 weeks if minor instability or pain is present, although most isolated cervical TPs, especially at C7, do not require bracing. While some athletes report psychological reassurance with bracing, it is not routinely needed.

### Indications for Surgery

**Fig. 10:**
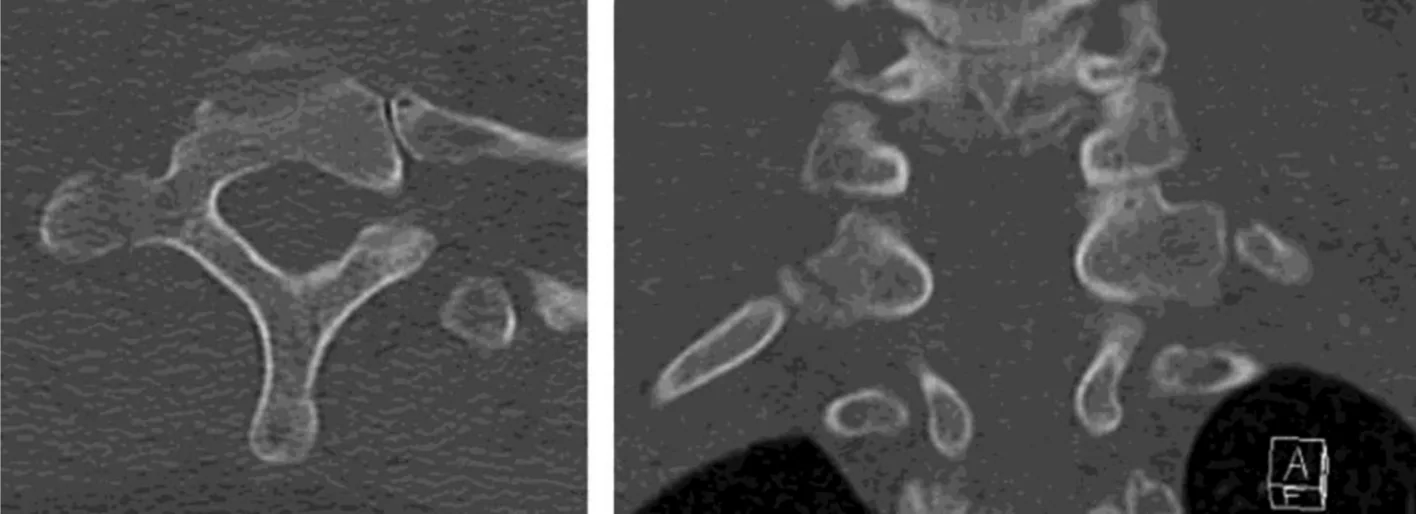
Axial and coronal CT scans of an isolated thoracic transverse process fracture [56]; reproduced from Peterson et al. [56], with permission

**Fig. 11:**
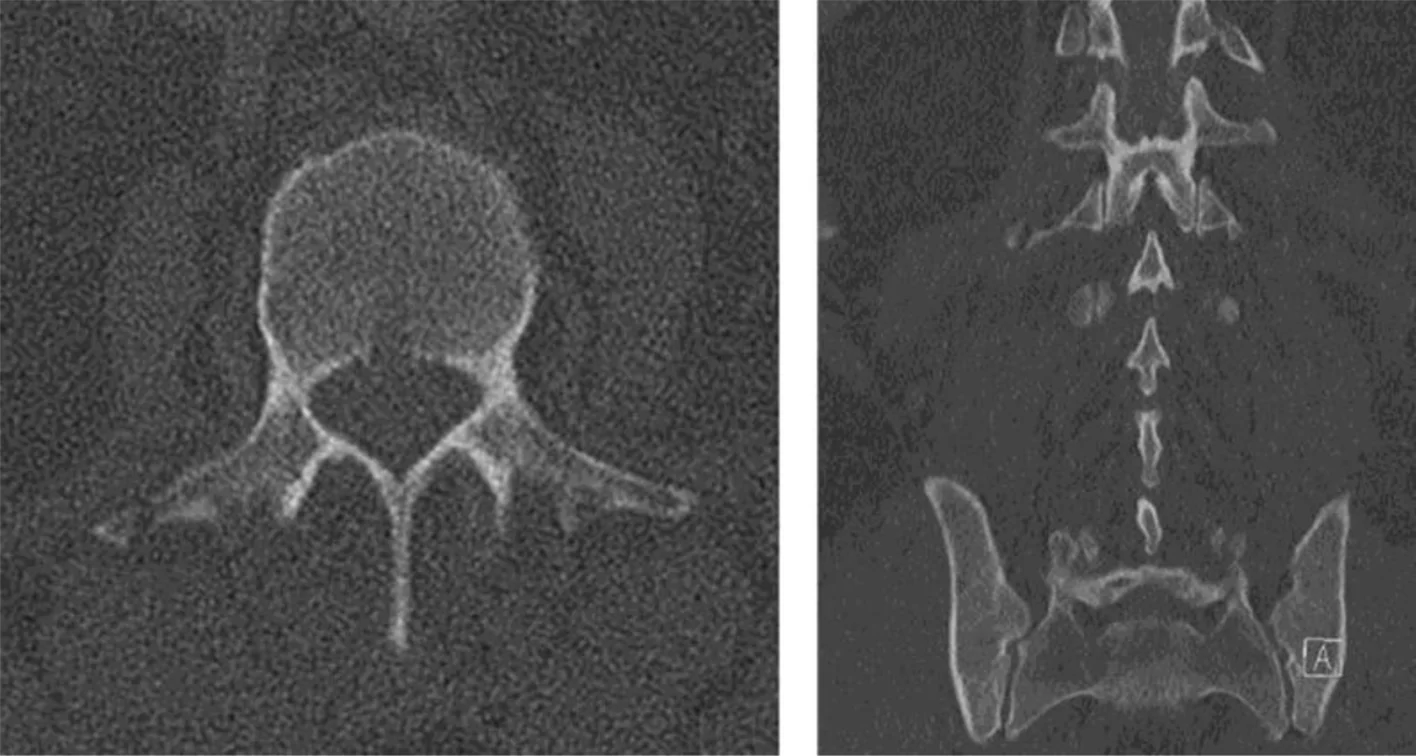
Axial and coronal CT scans of an isolated lumbar transverse process fracture [56]; reproduced from Peterson et al. [56], with permission

**Fig. 12:**
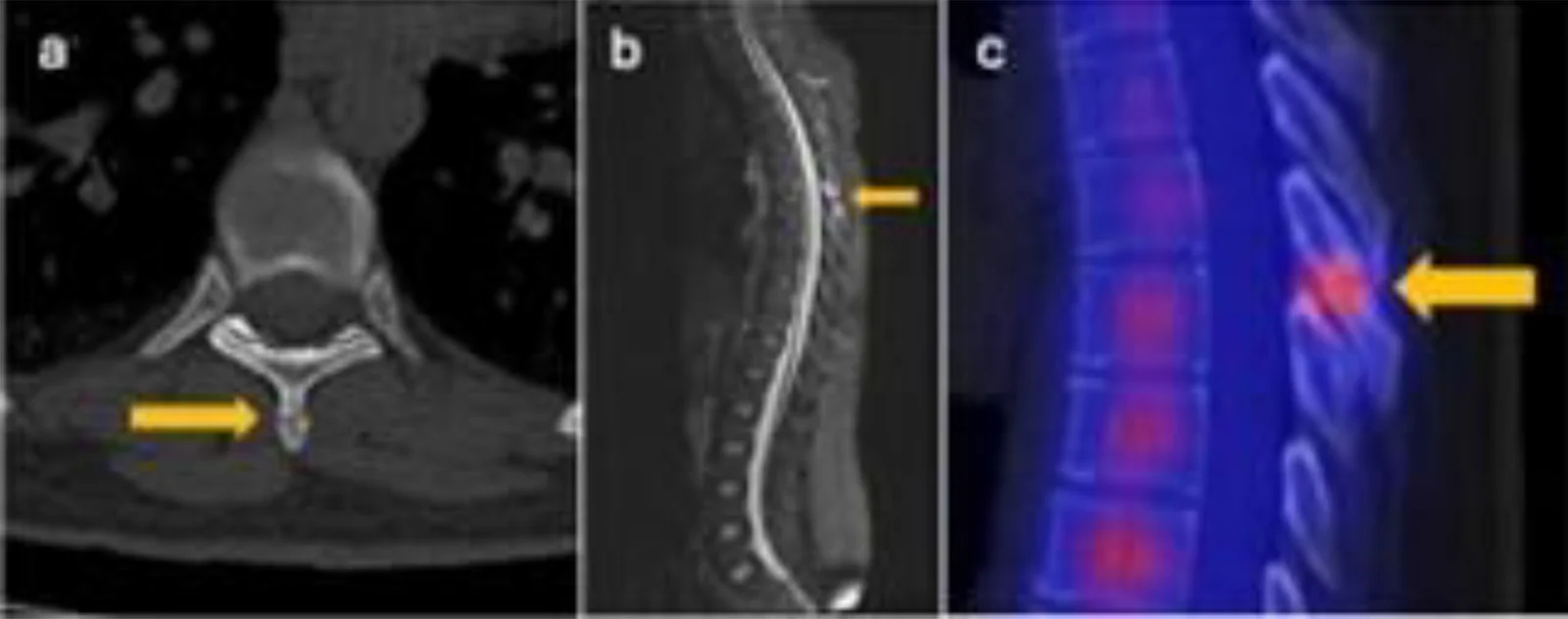
(**a**) CT, (**b**) short tau inversion recovery (STIR) sequence MRI and (**c**) single-photon emission computed tomography (SPECT) showing T6 spinous process non-union (defect indicated by yellow arrows) [60]; reproduced from Dietrich et al. [60], with permission

Surgical management of isolated TPFs is exceedingly rare. These fractures, by definition, do not destabilize the spine, and thus do not require fusion or fixation. A study conducted by Peterson et al. focussed on the clinical characteristics of isolated thoracic and lumbar TPFs and noted that these are stable injuries which do not require surgical intervention (Figs. 10 and 11) [56]. Surgery may be considered only in the context of complex injuries, such as unstable pedicle or facet fractures, or concurrent pelvic injury cases that extend beyond isolated TPFs. Rarely, painful non-union or symptomatic pseudoarthrosis may necessitate fragment excision (an example of a spinous process non-union fracture can be seen in Fig. 12) [13, 60]. Another surgical indication includes myositis ossificans formation at the TPF site, refractory to non-operative measures [13, 61]. Nonetheless, surgery is generally discouraged unless pain persists beyond the normal healing period, as fibrous tissue often stabilizes the fragment naturally, and surgical excision carries morbidity due to muscle dissection. A 2008 series involving 84 TPF cases found that surgical consultation was rarely necessary, reaffirming that these injuries are best managed non-operatively [10].

### Adjunct Therapies for Bone Healing

Elite athletes often use certain adjuncts to aid fracture healing. low-intensity pulsed ultrasound (LIPUS) has been used in clinical practice to stimulate fracture repair in certain fractures (e.g. tibial stress fractures, non-unions), by enhancing blood flow and callus formation [62]. LIPUS is non-invasive and safe, so it can be used daily for 20 min over the fracture site. Electrical bone stimulation using a pulsed electromagnetic field (PEMF) stimulator, which has shown promise in the treatment of long bone non-unions, can also be used to improve bone healing [63]. As mentioned in Sect. 5.1, stress fractures of the TP may develop in certain athletes. Since stress fractures are correlated with decreased metabolic function, nutritional optimization of the athlete may play a role in preventing TPFs [64]. On the basis of the guidance of qualified sports nutritionists, appropriate supplementation should be provided to correct any deficiencies and improve bone health (e.g. vitamin D).

Other physical modalities, such as soft tissue therapies, may also be utilized in the early stages of recovery, although the evidence supporting their use is very limited [13]. Some studies have noted that excessive direct deep tissue work on the fracture site, which is often combined with spinal manipulation, can lead to worsening neurological symptoms and soft tissue trauma [65]. Hence, they may be commenced only once some degree of tissue healing has occurred. In cases of persistent pain, interventional procedures such as image-guided corticosteroid or anaesthetic injections near the fracture site may be used, ensuring adherence to anti-doping principles on the use of corticosteroid injections in-competition [66, 67].

### Summary

Non-operative management is effective for TPFs. A period of modified load, analgesia, muscle relaxants and early mobilization form the foundation. No bracing is needed in most cases, and surgery is rarely required for the fracture itself. Adjunct therapies, such as bone stimulators and optimal nutrition, can be employed to facilitate healing, although natural healing is expected. The goal is to control pain enough to allow the athlete to start rehabilitation activities as soon as possible, since prolonged inactivity can be detrimental to athletic conditioning.

## Rehabilitation Following Transverse Process Fractures (TPFs)

Rehabilitation after a TPF in athletes should be structured, progressive and function oriented. As TPFs are stable injuries, rehabilitation can proceed based on pain tolerance, focusing on restoring spinal mobility, preventing deconditioning and enabling a safe, timely return to sport without placing early stress on the healing bone.

### Early Stage

The initial rehabilitation phase overlaps with acute management and emphasizes pain reduction, protection and gentle mobilization. Athletes are encouraged to begin deep breathing exercises immediately to prevent pulmonary complications. Ambulation (e.g. walking) should begin as tolerated to reduce deconditioning risks. Physical therapists may incorporate modalities such as ice, electrotherapy and light massage to the surrounding musculature, avoiding direct pressure on the fracture site.

As pain improves, therapists guide gentle spinal range of motion (ROM) exercises, tailored to movement restrictions. Early ROM helps prevent scar tissue formation and promotes flexibility. Movements, such as lateral bending and rotation—particularly away from the injured side—are often restricted until further healing occurs. Complete bed rest is discouraged; early mobilization paired with appropriate analgesia correlates with better outcomes [11, 13, 14]. Over several weeks, pain subsides and basic functional mobility improves significantly.

### Mid-stage

With pain reduction, active rehabilitation intensifies. Spinal flexibility and segmental mobility exercises are introduced, including light thoracic joint mobilizations (grade I–II), avoiding direct manipulation of the fracture site. By week 4, the goal is near-normal, pain-free ROM across all planes.

Once mobility is restored and pain levels decline, athletes begin progressive core and lower limb strengthening, alongside low-impact cardiovascular activities (e.g. cycling, swimming, elliptical training). These help reverse deconditioning from earlier inactivity. By weeks 4–6, most athletes can initiate advanced core work such as full planks and resisted trunk rotations. During this phase, soft tissue techniques including high-velocity thrusts (HVTs) may be applied carefully for pain relief, avoiding fracture destabilization. By the end of this stage, the fracture should be either fully healed or fibrously united, with minimal pain and nearly full ROM.

### Late Stage

#### Return-to-Play Readiness

This stage focuses on sport-specific reconditioning. Athletes progressively reintegrate dynamic core exercises (e.g. rotational medicine ball drills), weightlifting with strict form and modified sports participation. Sport-specific drills (e.g. dry swings for baseball, dribbling and shooting for soccer) are introduced gradually. Emphasis is placed on proper biomechanics to avoid compensation and reinjury. This phase also targets residual asymmetries or muscular imbalances using unilateral strengthening and targeted stretching. Teaching safe movement patterns and spinal protection techniques is critical.

Overcoming psychological barriers is equally vital. Gradual exposure to contact, often supported by sports psychologists, builds confidence. Protective gear (e.g. padded undershirts) may provide reassurance. Mental imagery techniques can also reduce fear-avoidance behaviours and support full psychological recovery.

#### Physical Therapy and Team Approach

Early physical therapy engagement is essential, as studies show isolated TPFs rarely require spine specialist input [10]. Effective communication with coaches helps tailor training loads appropriately. Throughout recovery, athletes should maintain cardiovascular and lower limb conditioning where possible to minimize overall deconditioning.

### Summary

TPF rehabilitation follows a logical, staged approach—from protection and gentle mobilization to sport simulation and full return. Pain, not structural instability, is the main limiting factor; if it is respected, athletes can advance activities appropriately without risking delayed healing.

## Relative Energy Deficiency in Sport (RED-S) and Its Role in TPFs

Relative energy deficiency in sport (RED-S) is an expanded framework of the Female Athlete Triad and refers to a state of chronic low energy availability that impacts menstrual function, bone health and other physiological systems affecting both women and men. In female athletes, RED-S commonly presents as menstrual dysfunction (oligomenorrhea or amenorrhea) and decreased bone mineral density (BMD), both of which contribute to increased risk of bone stress injuries, including stress reactions and fractures. The condition results from insufficient caloric intake combined with high energy expenditure, leading to hormonal changes such as hypoestrogenism and elevated cortisol levels. These hormonal disruptions impair bone remodelling and mineralization. Consequently, female athletes with RED-S are significantly more likely to sustain stress fractures compared with their eumenorrheic peers [64, 68]. Therefore, any low-impact or overuse fracture, such as a TPF without major trauma, should raise suspicion for underlying RED-S. Early identification through screening for menstrual irregularities, dietary patterns and bone health is essential to facilitate interventions that improve bone density and reduce injury risk.

### Risk Factors

The key risk factor in RED-S is low energy availability, which disrupts both hormonal and nutritional balance. Hypoestrogenism impairs calcium absorption and bone formation, contributing to low BMD. Athletes with RED-S often exhibit low BMD *Z*-scores at the spine or whole-body level, which correlates with a heightened risk of stress fractures [69]. Amenorrhea is both a marker and a mediator of compromised bone health. Low body mass index (BMI), particularly during periods of intense training, further exacerbates energy deficits and bone weakening. Nutritional deficiencies—especially in calcium and vitamin D—are common and contribute to impaired bone mineralization. For this reason, supplementation is recommended in high-risk individuals to prevent stress injuries. Clinicians should include RED-S in the differential diagnosis when a female athlete presents with persistent low back pain and a suspected TPF, and initiate prompt evaluation for energy imbalance, dietary deficits and menstrual dysfunction to guide appropriate management.

### Bone Health and Recovery Considerations

A TPF in a young female athlete should trigger a comprehensive assessment of bone health, particularly if the fracture occurs without significant trauma. Given the strong link between RED-S and bone fragility, clinicians should evaluate for menstrual history, dietary patterns and low BMD. Identifying a metabolic contributor is key to preventing recurrence. For example, in the reported case of a female rower with RED-S, investigations included evaluation of vitamin D and hormone levels and a dual-energy X-ray absorptiometry (DEXA) scan [11]. Nevertheless, most TPFs, when not associated with spinal instability, can be managed non-operatively with relative offload, pain control and early mobilization [70]. Bracing is typically unnecessary and may hinder recovery if overused [49, 59].

Healing timelines average 2–3 months, with an approximate RTP time of 9 weeks. In the rower’s case, she resumed low-impact training within 1 week, began light rowing by week 7 and was cleared for full training by week 13 [11]. This slightly extended recovery reflects the added metabolic burden of RED-S. In such cases, bone healing depends not only on rest but also on restored energy availability, adequate intake of calcium, vitamin D, protein and reduction in training load to allow remodelling.

Multidisciplinary care is crucial. Orthopaedic management should be complemented by sports medicine, nutrition, endocrinology, gynaecology and psychological support. Addressing amenorrhea through increased caloric intake or reduced training, and treating any disordered eating behaviours, are essential for long-term bone and athletic health. Ultimately, management of a TPF in the context of RED-S extends beyond fracture healing: it necessitates a comprehensive, systemic approach to restore bone integrity and prevent future injuries.

## Future Directions in Research and Prevention

An increased awareness of bone stress injuries, particularly TPFs, has driven efforts to improve clinical outcomes and reduce the occurrence of such injuries. Recent efforts have focused on building expert consensus and leveraging new technologies to guide clinical decision-making:

### Consensus Return-to-Play Guidelines

Until recently, there was little international agreement on how to define and manage bone stress injuries. In response, experts conducted an international Delphi study in 2025, which reached consensus on diagnosis, risk assessment and return-to-play (RTP) guidelines and outlined a multifactorial approach to identify and manage TPFs while promoting bone health [71]. This initiative provided recommendations in areas where evidence had been sparse, highlighted knowledge gaps and set a research agenda. The resulting consensus offers clinicians structured recommendations to support safer RTP decisions and more consistent care across settings.

### Improved MRI and Diagnostic Tools

Advances in imaging are enhancing our ability to detect and monitor bone stress injuries, which is particularly valuable for early intervention. MRI detects stress injuries earlier than an X-ray and remains the gold standard. Newer MRI sequences and grading scales are refining this capability. Fluid-sensitive sequences (such as STIR) can detect minor acute bone injuries early and enable prompt diagnosis before a complete fracture develops [40]. Research has shown that MRI-based injury grades correlate with clinical severity and can predict RTP duration, aiding in prognosis. Other advanced MRI techniques, such as UTE MRI, which can more accurately track bone healing, have paved the way for individualized rehabilitation planning which would enable timely RTP [41].

### Injury Prevention and Management Focus

There is growing emphasis on preventing bone stress injuries through education, training modification and proactive monitoring. Screening initiatives can identify risk factors related to training, biomechanics and nutrition to enable early identification and intervention. Guidelines recommend that athletes maintain energy balance and adequate intake of bone-supportive nutrients such as calcium, vitamin D and protein, while avoiding further caloric restriction. Training programs are also being adjusted to prioritize bone health, by limiting rapid increases in training volume and allowing adequate rest. Moreover, multidisciplinary care models, combining the physician, dietitian, physiotherapist and mental health professional, can enable the early modification of underlying risk factors. Such approaches focus on the athlete as a whole and aim to break the cycle of repeated injuries. Future research will likely investigate the efficacy of specific interventions such as nutritional supplementation, hormonal therapies and pharmacological options for speeding up fracture healing and restoring bone density in athletes predisposed to stress injuries.

### Summary

The management of bone stress injuries, particularly TPFs, is shifting towards a more holistic and evidence-based model. Continued research will further clarify how best to protect bone health in at-risk athletes and safely return them to play after injuries. The goal is to reduce the incidence of injuries, such as TPFs, by addressing the root causes and ensuring that susceptible athletes can train and compete safely while maintaining robust bone health.

## Risks Associated with the Injury

While TPFs generally heal well, there are certain risks and potential consequences to be mindful of, especially in the context of athletic careers. These can be categorized into short-term risks (e.g. playing too soon, managing a ‘stable’ fracture under competitive pressures) and long-term considerations (e.g. what if the fracture does not heal perfectly, or if there are repetitive injuries).

### Risks of Playing Through a ‘Stable’ TPF

Athletes and teams sometimes contemplate whether a player can continue playing or return very quickly, given that the fracture is stable. Some players may use local anaesthetic injections to make an early RTP following rib injuries [72]. However, high-impact activity on a fresh fracture can not only worsen the injury, but also prolong recovery owing to an increased risk of developing additional complicated injuries. Additional injuries may be a result of repeated damage to the fractured segment, or owing to muscular imbalances resulting from involuntary compensatory mechanisms [73]. Repetitive use of a fractured segment without proper healing can also cause complications, such as non-union of the fracture segments or myositis ossificans, which may cause ongoing pain and require surgical removal [60, 61]. These complications have been noted in numerous studies [3, 12, 13].

### Long-Term Consequences

The long-term consequences of TPFs can be due to either physiological factors or improper management. Physiologically, residual pain, non-union, pseudoarthroses or early osteoarthritis can occur. Although chronic pain is not a common consequence of TPFs, such as disc herniations or pars fractures, it may occur [3, 74]. It can occur owing to fibrous non-union of fracture segments, which has been reported in the literature and has been shown to cause residual pain [12, 75]. Malunion of the fracture segments may also lead to pseudoarthroses, which might irritate surrounding tissues. These abnormal healing sequences warrant careful follow-up. The bone trauma experienced during TPFs may also predispose the bone to early localized osteoarthritis. Although there is a paucity of literature correlating TPFs to osteoarthritis, studies have shown that pro-inflammatory cytokines released into the joint after injury can predispose the joint to osteoarthritis [76]. Improper management of the athlete’s physical and mental health can not only cause non-union but may also lead to psychological fears of reinjury [77]. Thus, measures should be taken to ensure complete physical and mental recovery after injury.

### Playing with Undiagnosed TPF

The case of the high school American Football player, outlined by Brynin et al., underscores the danger of a missed diagnosis [13]. He continued to play and received manipulative therapy while the fracture was present, which could have had catastrophic neurological consequences (e.g. causing neurological injury if there had been an undetected instability) [78]. This sheds light on the idea that the improper assessment of an athlete with back pain can lead to the misdiagnosis of underlying spinal injuries and other insidious pathologies that are often present in association with this fracture. This could have negative systemic consequences. Modern protocols therefore err on the side of thorough evaluation—for instance, any athlete with significant back trauma, such as a compression or burst fracture, is often put through a concussion-style protocol for the spine: they must be pain-free, have full ROM and no neurological deficits before being allowed back in the same game, or they sit out pending imaging [79]. If an athlete has a relatively minor fracture, such as a spinous or TPF, serial imaging is not usually required if clinical criteria are met.

### Risk–Benefit of Early Return

In professional sports, decisions may ‘push the envelope’ for returning players quickly (for playoffs, etc.). Many professional teams have allowed athletes to return very rapidly from TPFs, seemingly without major incident. This suggests that with careful management, early return is possible, but it is a calculated risk balancing pain tolerance and re-injury chance and athletes should be at the centre of informed decision-making in these circumstances. The risk of playing through pain is primarily that the athlete underperforms or worsens the pain, whereas the risk of creating a worse structural injury is relatively low if the spine is stable. Each case should be individualized; generally, if an athlete has pain under reasonable control, no other injuries and can perform required movements, some might clear them earlier than the typical 3–4 weeks. However, doing so always carries the caveat that if pain worsens or function degrades, they must be withdrawn and re-evaluated.

## Return-to-Play Considerations

Determining when a professional athlete is ready to RTP after a TPF requires a combination of clinical assessment, sport-specific testing, and imaging in certain cases. The overarching principle is that the athlete should have achieved healing on the basis of sound clinical decision-making, sufficient to protect them from further injury and allow effective performance. Additionally, any associated injuries must have resolved to an acceptable degree. Below are key RTP criteria and considerations, followed by examples from case studies.

### Medical Clearance Criteria

Most sports medicine physicians would require certain criteria to be met before clearing an athlete with a TPF. This includes absence of significant pain, full neurological function, restored flexibility and strength and psychological readiness [79, 80]. Pain should be minimal and not impair function and should not require more than minimal analgesia. Specifically, full spine range of motion and sporting function should be achievable without more than mild discomfort. For instance, a lumbar TPF athlete should be able to flex, extend, rotate and laterally bend the trunk fully without sharp pain. No neurological symptoms should be present, and a neurological examination must be normal. Any neurological issues would delay return until resolved and perhaps necessitate further evaluation. Furthermore, the athlete should have regained pre-injury levels of flexibility. Functional tests (mimicking sport-specific movements) can be employed to assess the degree of symmetry and compensation. Isometric abdominal muscle tests (planks, side planks) might also be used to ensure sufficient pain-free trunk mobility and strength.

In addition to the above, the athlete must demonstrate the ability to perform sport-specific activities at match intensity. This often involves a gradual reintroduction to full practice [11, 14]. They should be able to get through rigorous practice sessions including any contact elements equivalent to game situations before actual competition. If a player can practice fully with the team for consecutive sessions with no setbacks, that is a strong sign they are ready. If applicable, the athlete should have and be acclimated to any protective gear (such as a flak jacket) that will be used in games.

### Imaging for Clearance

Radiographic healing provides reassurance but is not always mandatory if clinical criteria are met [79]. In cases of spondylolysis or spinous and TPFs, serial imaging studies are usually not recommended. However, in some cases an MRI (including 3D VIBE sequences) or CT might be obtained around the RTP decision point to assess the level of tissue healing. This is because these imaging modalities can highlight anatomical subtleties that are associated with the risk of reinjury [48]. However, as previously noted for TPFs, the absence of complete bony union is not an absolute contraindication to RTP if the athlete is symptom-free and functional [3, 12]. The decision is often individualized and based on resource and context: if any doubt exists, imaging helps; if clinical markers are reassuring, imaging may not be required.

### Case Studies of Return-to-Play

In the NFL series by Tewes, players with TPFs on average missed 3.5 weeks [12]. They often wore padding on return, and as noted, only one had a recurrence. Similarly, in the case presented by Brynin et al., the player had a gradual RTP, with the first game played at 4 weeks after injury using a back pad [13]. These cases suggest that an aggressive RTP (under a month) can succeed in cases where the athletes meet the above criteria. An increased time to RTP could be a consequence of complicated injuries or a cautious management approach. In the case of the fast bowler, the time to complete RTP was 9–10 months, with the player advised to play with a spinal brace for the first 5 months [3]. This was understandable since the patient had fracture non-union of the left L1–L5 TPs. The earlier mentioned soccer player returned at ~ 10 weeks, which was likely a consequence of a cautious approach employed owing to the time (mid-season) and nature (multi-level) of the injury [14]. McGuire et al. provided insight into the investigations which should be performed in the case of suspected non-traumatic TPFs in susceptible athletes [11]. The authors initiated a gradual RTP program with light activities commenced at 7 weeks and a full RTP at 13 weeks. Furthermore, they conducted a metabolic workup and DEXA scan to rule out pathophysiological causes of the fracture.

### Preventing Re-Injury

On returning, athletes should continue with core strengthening exercises and wear protection if required. However, realistically, one cannot fully prevent an unusual impact. The best prevention is making sure the body is as robust as possible—hence the focus on rehabilitation to not only heal the bone but also the supporting muscles.

### Summary

**Fig. 13:**
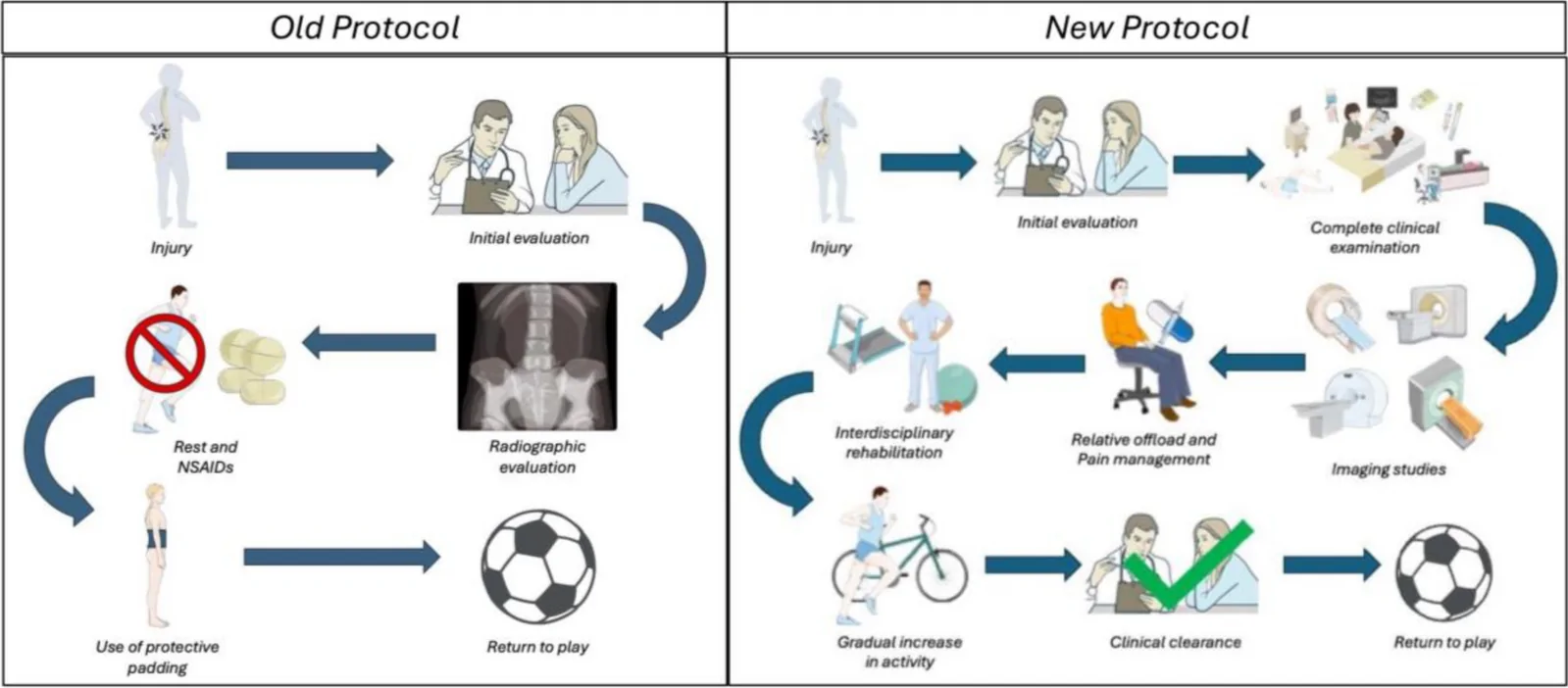
A figure comparing the original and revised treatment recommendations for the management of transverse process fractures [21, 81, 82]

In conclusion, an athlete can be cleared to RTP after a TPF when they have no significant pain, full functional range of motion and strength, are psychologically ready, have completed a graded RTP including sport-specific contact and when any imaging or medical considerations are satisfactory. Each case may vary slightly, but these principles ensure that the athlete’s return is safe and that they can perform at their expected level. With these criteria met, most athletes successfully return to their sport without limitations, as evidenced by numerous case studies of professionals resuming high-level competition following TPFs. The sports physician’s role is to balance a timely return with thorough safety checks, and to communicate accurately with all stakeholders to ensure well-rounded, holistic care for the athlete—given the generally benign nature of TPFs, this balance can usually be struck such that both the athlete’s health and competitive interests are served. An illustration explaining these treatment guidelines is shown as Fig. 13.

## Conclusions

TPFs, although stable, warrant careful evaluation and management in professional athletes owing to functional demands. While isolated TPFs do not compromise spinal stability, their functional implications—especially in sports requiring rotational or high-impact movements—necessitate a structured, individualized approach to rehabilitation and RTP. The prognosis is generally favourable, with most athletes resuming competition within weeks, provided there is no associated injury or significant pain limitation.

Key considerations in TPF management include ruling out concomitant injuries, optimizing pain control and initiating early mobilization to prevent deconditioning. Imaging, particularly CT, plays a crucial role in diagnosis, while MRI (with 3D VIBE sequences) may be valuable in assessing soft tissue involvement or stress-related injuries. Non-operative treatment remains the cornerstone, with NSAIDs, muscle relaxants and activity modification forming the foundation of early care. Bracing is rarely necessary, and surgical intervention is almost never indicated.

RTP decisions should be guided by resolution of pain, restoration of full spinal mobility and the athlete’s ability to perform sport-specific movements without compensation. The presence of multi-level fractures, non-union or persistent functional deficits represent poor prognostic signs. Ultimately, TPFs exemplify the balance required in sports medicine—prioritizing athlete safety while facilitating an expedient RTP. A multidisciplinary approach involving physicians, physiotherapists, sport science and coaching staff should thus be employed. With appropriate management, most athletes with TPFs achieve complete recovery and resume high-level performance without significant risk of recurrence or persistent dysfunction.

## Declarations

### Availability of data and material

All data supporting the findings of this study can be found in the manuscript. All figures included in the study are either original works of the authors or adapted from open access studies after obtaining consent from relevant parties.

### Code availability

No software applications were used in this study.
